# Effects of SWEEPS-Activated Irrigation and Other Methods for Elimination of Intracanal Medicaments on Push-Out Bond Strength of NeoMTA2 to Root Dentin: An In Vitro Study

**DOI:** 10.1155/tswj/7246588

**Published:** 2025-02-10

**Authors:** Maryam Babaahmadi, Fatemeh Dibaji, Mehdi Vatanpour, Mohsen Aminsobhani, Pegah Sarraf, Mehrfam Khoshkhounejad

**Affiliations:** ^1^Department of Endodontics, School of Dentistry, Tehran University of Medical Sciences (TUMS), Tehran, Iran; ^2^Department of Endodontics, School of Dentistry, Islamic Azad University Tehran, Tehran, Iran; ^3^Department of Endodontics, Dental Research Center, AJA and Tehran University of Medical Sciences, Tehran, Iran; ^4^Dental Research Center, Dentistry Research Institute, Tehran University of Medical Sciences, Tehran, Iran

**Keywords:** calcium hydroxide, calcium silicate, Er YAG laser, push-out bond strength, root canal medicament, therapeutic irrigation

## Abstract

**Objectives:** This study evaluated the effects of laser-assisted irrigation, conventional syringe irrigation (CSI), and passive ultrasonic irrigation (PUI) for elimination of intracanal medicaments on push-out bond strength (PBS) of NeoMTA2 to root dentin.

**Materials and Methods:** In this in vitro study, 150 extracted single-rooted mandibular premolars were decoronated and standardized with a certain root length. The canals were instrumented to simulate immature roots and randomly assigned to three experimental groups (*n* = 45) using either triple antibiotic paste (TAP), double antibiotic paste (DAP), or calcium hydroxide (CH) as intracanal medicament and one no-medicament control group (*n* = 15). After 28 days, the experimental groups were randomly divided into three subgroups (*n* = 15) according to the irrigation method using either erbium-doped yttrium aluminum garnet (Er:YAG) laser shockwave-enhanced emission photoacoustic streaming (SWEEPS), CSI, or PUI. A dentinal ring was then obtained from the coronal part of each root, and its lumen was densely filled with NeoMTA2. After 1 week, the PBS was measured using a universal testing machine. Data were analyzed by ANOVA and Tukey and Dunnett tests (alpha = 0.05).

**Results:** The interaction effect of the irrigation technique and medicament type on PBS was significant (*p* < 0.05). The PBS in all groups was significantly lower than the control group (*p* < 0.01) except in CH-SWEEPS (*p* = 0.741). In the experimental groups, the PBS of the SWEEPS subgroup was significantly higher than other subgroups (*p* < 0.001). The PBS of PUI was higher than CSI in CH and TAP groups (*p* < 0.001). The PBS of CH was significantly higher than TAP in CSI, and DAP and TAP in PUI and SWEEPS groups (*p* < 0.05).

**Conclusion:** In this in vitro study, regardless of the irrigation method, higher PBS of NeoMTA2 to root dentin was achieved in CH groups compared to TAP. A higher PBS was also achieved when SWEEPS and PUI methods were used to eliminate CH in comparison to TAP and DAP.

## 1. Introduction

Root canal disinfection is a critical step in regenerative endodontic treatment (RET) of immature necrotic teeth with thin dentinal walls and open apices [1]. RETs are performed to regenerate a functional dentin-pulp complex in immature teeth, enable apex closure, and reinforce thin root canal walls by increasing the length and thickness of dentin [2, 3]. In RETs, the root canal space is disinfected mainly by using root canal irrigants and intracanal medicaments [4].

Triple antibiotic paste (TAP), which is the most commonly used medicament for root canal disinfection in RETs [5], consists of equal amounts of metronidazole, ciprofloxacin, and minocycline [5]. To avoid minocycline-induced coronal discoloration, many researchers suggested the application of double antibiotic paste (DAP), which includes equal amounts of metronidazole and ciprofloxacin [6]. Calcium hydroxide (CH) is another commonly used intracanal medicament for root canal disinfection in RETs, which has a high success rate [7]. In addition to efficient root canal disinfection, success of RETs depends on establishment of a hermetic coronal seal to prevent bacterial penetration [4]. Calcium silicate (CS)–based cements have been successfully used as a coronal barrier for sealing of the root canal space and prevention of bacterial leakage [8].

Effective elimination of intracanal medicaments is another challenge in RETs as remnants of TAP, DAP, and CH have shown to have some toxic effects on stem cells [1, 9] and also decrease the bond strength of some CS-based cements to root dentin [8, 10–12].

The effect of residual intracanal medicaments in the root canal system on push-out bond strength (PBS) of CS-based cements has been previously evaluated, and it has been demonstrated that cleaner dentin surfaces in contact with CS-based cements result in a higher PBS [13, 14]. However, the results have been controversial in this regard [2, 8, 10, 15]. It may be speculated that a more efficient method for removal of intracanal medicaments may indirectly improve the PBS of CS-based cements to root dentin.

It has been demonstrated that the application of root canal irrigants alone is not sufficient for complete removal of TAP and even DAP from the root canal system [7, 16]. Some other studies showed inefficacy of different root canal irrigation protocols, such as conventional syringe irrigation (CSI), passive ultrasonic irrigation (PUI), and EDDY for complete elimination of CH from the root canal system [17–19]. The irregularities and complexities of the root canal system can make the effective removal of medicaments even more challenging in clinical settings. Some authors have tried to simulate these irregularities by making artificial grooves and evaluate the efficacy of different removal protocols [20, 21]. Arslan found that without ultrasonic activation it was difficult to remove TAP from artificial grooves in dentin [16].

Thus, several different methods have been proposed for the elimination of intracanal medicaments from the root canal system [17, 18, 22]. Positive-pressure CSI is the simplest and most common method for this purpose [7]. PUI has been introduced as a method for activation of irrigants and enhancement of their efficacy for the elimination of root canal debris and microorganisms [23]. Photon-activated irrigation is another technique for agitation of irrigants and improvement of their efficacy [24]. It causes a photon-induced photoacoustic streaming (PIPS) in the irrigant, which leads to acoustic agitation and enhancement of cleaning efficiency [24]. A previous study showed the superiority of this technique to CSI and PUI in removal of TAP and DAP residues from the root canal irregularities [21]. The shockwave-enhanced emission photoacoustic streaming (SWEEPS) technique is a new laser modality suggested for this purpose, which has showed promising results in the elimination of CH from the root canal system [22].

No consensus exists regarding the most efficient method for the elimination of medicaments from the root canal system in RETs. Also, to the best of the authors' knowledge, no previous study has used SWEEPS endodontic laser treatment for the elimination of TAP and DAP from the root canal space. Thus, this study aimed to assess the effects of CSI, PUI, and erbium-doped yttrium aluminum garnet (Er:YAG) laser in the SWEEPS mode for the elimination of TAP, DAP, and CH on PBS of NeoMTA2 (Avalon Biomed, USA) CS-based cement to root dentin. The null hypothesis of the study was that no significant difference would be found in the effects of different irrigation protocols on PBS of NeoMTA2 to root dentin.

## 2. Materials and Methods

This in vitro study was conducted on 150 caries-free mature single-rooted human mandibular premolars extracted within the past 3 months due to periodontal problems or as part of the orthodontic treatment. The teeth had almost the same root length and diameter. The study protocol was approved by the ethics committee of the university (IR.TUMS.BLC.1402.060).

### 2.1. Sample Size

The minimum sample size was calculated to be 15 in each group using one-way ANOVA power analysis of PASS 11 and according to a previous study [13] assuming alpha = 0.05, beta = 0.1, mean standard deviation of PBS to be 30 megapascals (MPa), and effect size of 0.38.

### 2.2. Specimen Preparation

Initial preparation of the teeth was performed according to Alsubait et al. [13]. The teeth were disinfected by immersion in 0.5% chloramine T solution for 1 week. Soft tissue and calculus were removed from the root surfaces by using a periodontal scaler. All teeth were inspected under a dental operating microscope (F170; Carl Zeiss, Germany) at x4 magnification, and those with root caries, cracks, or fracture were excluded. The teeth were then radiographed from the mesiodistal and buccolingual directions to ensure the presence of one single canal with no calcification and no evidence of internal root resorption. Also, roots with a curvature > 20° were excluded [25]. The teeth were stored in saline at 4°C until the experiment.

The teeth were then decoronated at 2 mm above the cementoenamel junction (CEJ) of their buccal surface and were also sectioned at 12 mm below their buccal CEJ by using a diamond disc (Microdont, Brazil) under water coolant to standardize the root length. In order to standardize the diameters of root canals, the teeth with root canals wider than a # 25-K-file were excluded from the samples. Working length was determined by inserting a #15-K-file (Maillefer, Ballaigues, Switzerland) in the canal until the tip was visible at the apical foramen. A glide path was established by using a #15-hand K-file to the working length, and the root canals were then prepared by ProTaper rotary files (Dentsply Maillefer, Ballaigues, Switzerland) up to F5 at a speed of 300 rpm. Peeso reamers #1 to #5 (Mani Inc., Tochigi, Japan) were used to the working length to parallelize the internal walls and standardize the root canal lumen diameter by 1.5 mm. Finally, #5 peeso reamer passed through the apex by 1 mm to simulate an open-apex tooth. After using each instrument, the root canals were irrigated with 2 mL of 2.5% NaOCl (Hypo Endox, Morvabon, Iran). The root canals were subsequently irrigated with 5 mL of 2.5% NaOCl and then 5 mL of 17% EDTA (Parla Co., Iran) each for 1 min for smear layer removal. A final rinse with 10 mL of distilled water was then performed for elimination of residual irrigants. The root canals were finally dried with sterile paper points (Meta, Korea). Irrigation of the root canals during the entire process was performed with a positive-pressure irrigation method using a 25-gauge closed-end double side-vented needle (Fanta, China). All specimens were prepared by the same operator under a dental operating microscope with x4 magnification. Then, the specimens were randomly assigned to three experimental groups (*n* = 45) for the application of TAP, DAP, or CH as intracanal medicament, and one control group (*n* = 15) with no intracanal medicament. The experimental groups were randomly assigned to three subgroups (*n* = 15) for elimination of intracanal medicaments with positive-pressure CSI, PUI, and Er:YAG laser (SWEEPS). In the experimental groups, the apex was sealed with glass ionomer cement (Fuji, GC, Tokyo, Japan) prior to the application of the medicaments.

TAP was prepared using equal (1:1:1 by weight ratio) amounts of metronidazole, ciprofloxacin, and minocycline powders obtained by crushing the tablets after removing their coating by a #15-scalpel (Moris, Germany). A suspension was prepared by mixing the required amounts of the powders with distilled water in 5 mg/mL concentration, which is the highest concentration recommended by the American Association of Endodontists for RETs [26].

DAP was prepared by using equal amounts (1:1 by weight ratio) of metronidazole and ciprofloxacin. A suspension was made by mixing the powders with distilled water in 5 mg/mL concentration. TAP and DAP were delivered into the canals by using a 23-gauge syringe.

CH intracanal medicament (Golchai, Iran) was prepared with a concentration of 16 mg/mL according to Khoshkhounejad et al. [9] by mixing CH powder with distilled water.

CH was delivered into the root canals by using a 23-gauge syringe. The canals were entirely filled with the medicaments to the CEJ (orifice). The penetration depth of the syringe was up to 1 mm to the working length. After the application of the medicaments, the access cavity was sealed with a sterile Teflon pellet (Dena Teflon, Iran) followed by the application of glass ionomer cement (Fuji, GC, Tokyo, Japan). The specimens were then incubated at 37°C and 95% humidity for 28 days.

### 2.3. Irrigation Protocols for Elimination of Intracanal Medicaments

After 28 days, the temporary coronal restoration of the specimens was removed by a high-speed handpiece under air and water spray, and the medicaments were removed by the following three methods in the three subgroups.

For positive-pressure CSI, a 25-gauge closed-end double side-vented needle (Fanta, China) was used for irrigation of the root canals with 20 mL of 17% EDTA for 2 min according to Eymirly et al. [19]. The needle was introduced into the canal passively (with no retention) up to 1 mm to the working length, and 5 mL of sterile saline was used as the final irrigant. The canals were then dried with paper points.

For PUI, samples were rinsed with 20 mL of 17% EDTA for 2 min by placement of the U ultrasonic tip of a #20-U-file (Eighteeth, Ultramint Pro, China) in the canal. For this purpose, after using 5 mL of EDTA, it was activated by the U tip of the ultrasonic instrument (endo mode, power of three) for 30 s such that the file tip was placed at 1 mm distance from the apex and had no contact with the canal walls. Three more episodes of irrigation with 5 mL of EDTA and activation by the ultrasonic instrument for 30 s was done (a total of 2 min), and then 5 mL of saline was used for the final rinse. The canals were subsequently dried with paper points.

In the laser subgroups, 2940 nm Er:YAG laser (Fonta, Slovenia) with the SWEEPS tip made of quartz was used in auto-SWEEPS mode. The outer diameter of laser probe was 1.1 mm, and for all samples, the energy level was set on 20 mJ with a frequency of 20 Hz and average power of 0.8 W. The air/water spray of the laser handpiece was off during the process. The canals were then irrigated with 5 mL of 17% EDTA with a 25-gauge closed-end double side-vented needle passively up to 1 mm to the working length. Then, the fiber optic was placed in the access cavity (without introducing into the canal) and activated for 30 s. Three more episodes of rinsing the canals with 5 mL of 17% EDTA which was subsequently activated by laser for 30 s was done. Accordingly, the whole activation time was 120 s, and 20 mL of 17% EDTA was used in total. After the fourth episode of activation, 5 mL of saline was used for the final rinse. The canals were then dried with paper points. As mentioned earlier, medicament application and removal were not performed for the control group.

### 2.4. Measurement of PBS

Twenty eight days after the application of the medicaments [27, 28], the temporary coronal restoration was removed by a round bur and high-speed handpiece under air/water coolant, the medicaments were removed as mentioned earlier, and the roots were horizontally sectioned by a low-speed saw under water coolant. Root sections (dentinal rings) with 2 ± 0.1 mm standard height were obtained from the coronal part of the roots [2, 13, 15, 29] 1 mm below the CEJ. The thickness of each slice (ring) was measured by a digital caliper (Mitutoyo, Japan) with 0.001 mm accuracy. Then, NeoMTA2 (Avalon Biomed, United States of America) was applied in the dentinal rings. It was mixed as instructed by the manufacturer, delivered into the lumen, and packed by using an endodontic plugger against a smooth glass slab. Excess cement was removed by a scalpel. The specimens were then wrapped in a gauze dipped in phosphate-buffered saline (Dacell, Iran) and incubated at 37°C for 1 week. Then, the PBS test was performed in a universal testing machine (Z050, Zwick Roell, Germany). For this purpose, the specimens were placed on a metal plate with a hole in the middle for free movement of the plunger, and load was applied apicocoronally to the cement surface in the lumen. The diameter of the plunger tip was 1.3 mm, and it applied a constant vertical load at a speed of 1 mm/minute until cement dislodgement. The plunger tip was adjusted such that it only contacted the cement surface. Maximum load causing cement dislodgement was calculated and recorded in MPa using the following formula:

(1) $$\begin{array}{c} \text{PBS }\left(\text{MPa}\right)=\frac{\text{Load required for cement dislodegment in Newtons}}{\text{Bonded surface area in square millimeters }}, \end{array}$$

where bonded surface area = 3.14 × 2 x root canal radius *x* dentinal ring thickness.

### 2.5. Failure Mode

**Figure 1:**
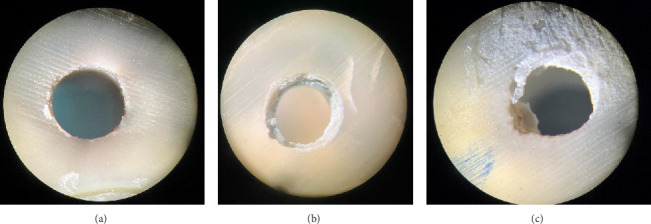
Images of various failure modes: (a) adhesive; (b) cohesive; (c) mixed, determined under stereomicroscope (SMZ800; Nikon, Japan) at × 30 magnification.

The failure mode was determined under a stereomicroscope (SMZ800; Nikon, Japan) at × 30 magnification and categorized as adhesive (debonding at the dentin–cement interface), cohesive (fracture within the cement), and mixed (a combination of both adhesive and cohesive) (Figure 1). The operator who inspected the specimens was blinded to their group allocation.

### 2.6. Statistical Analysis

Data were analyzed using two-way and one-way ANOVA. Pairwise comparisons were performed by Tukey's test (due to homogeneity of the variances as confirmed by Levene's test) and Dunnett's test. The level of statistical significance was set at 0.05.

## 3. Results

### 3.1. PBS

Table 1 presents the mean PBS of the study groups. Two-way ANOVA revealed a significant interaction effect of the irrigation method and type of medicament on PBS (*p* < 0.05). Thus, the effect of each irrigation method on PBS was independently analyzed by one-way ANOVA, followed by pairwise comparisons.

Comparison of the PBS of the control group with the experimental groups by Dunnett's test revealed that the PBS in all experimental groups was significantly lower than that in the control group (*p* < 0.01 for all) except the CH/SWEEPS group, which had no significant difference with the control group (*p* = 0.741).

Pairwise comparisons of the experimental subgroups based on the type of medicament revealed that in the CH and TAP groups, the PBS of the SWEEPS subgroup was significantly higher than the other two subgroups (*p* < 0.001). Also, the PBS of the PUI subgroup was significantly higher than that of the CSI subgroup (*p* < 0.001).

In the DAP group, the PBS of the SWEEPS subgroup was significantly higher than the other two subgroups (*p* < 0.001). However, the difference in PBS between the CSI and PUI subgroups was not significant (*p* = 0.076).

Comparisons of the groups based on the irrigation method revealed that in the CSI and PUI, the PBS of all three medicament groups was significantly lower than that of the control group (*p* < 0.001 for all). Pairwise comparisons of the medicaments revealed significantly higher PBS in the CH/CSI group than the TAP/CSI (*p* = 0.023). The difference in PBS was not significant between the CH/CSI and DAP/CSI (*p* = 0.93) or TAP/CSI and DAP/CSI (*p* = 0.054).

Significantly higher PBS of the CH/PUI than TAP/PUI (*p* = 0.003) and DAP/PUI groups (*p* = 0.007) was also revealed. The difference in PBS of TAP/PUI and DAP/PUI was not significant (*p* = 0.956).

**Figure 2:**
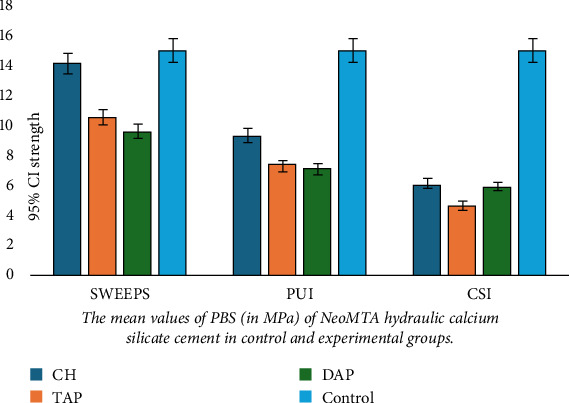
The mean values and standard deviations of PBS (in MPa) of NeoMTA2 hydraulic calcium silicate cement in control and experimental groups.

**Figure 3:**
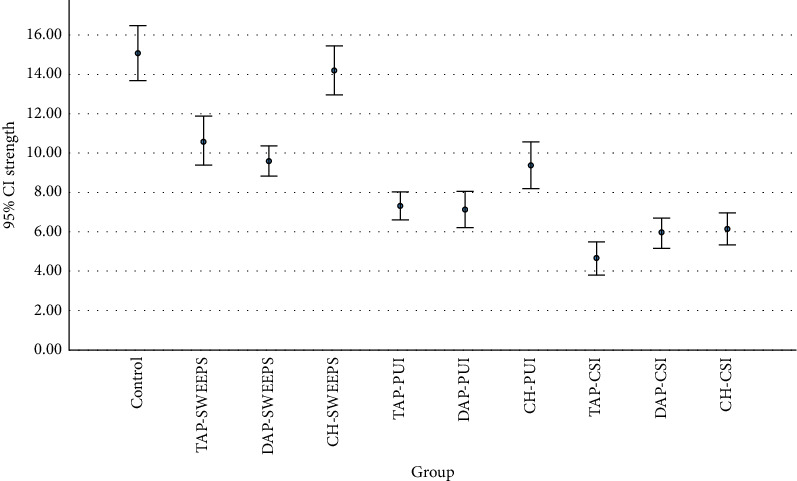
Error bars showing the means and standard deviations of PBS (in MPa) of NeoMTA2 hydraulic calcium silicate cement in control and experimental groups.

In the SWEEPS groups, the PBS of the CH and control groups was not significantly different (*p* = 0.741). However, the PBS of TAP/SWEEPS and DAP/SWEEPS was significantly lower than that of the control group and the CH/SWEEPS (*p* < 0.001 for both). The difference in PBS of TAP/SWEEPS and DAP/SWEEPS was not significant (*p* = 0.356). Figures 2 and 3 indicate the mean values of PBS (in MPa) of NeoMTA2 hydraulic CS cement in control and experimental groups.

### 3.2. Failure Mode

Table 2 presents the frequency of different failure modes in the control and experimental groups. In the control and CSI and PUI groups, the most frequent failure mode was mixed in approximately over 50% of the specimens while in the SWEEPS group, the most frequent mode of failure was cohesive. Adhesive failure was the least frequent in all the experimental and control groups. Also, adhesive failure did not occur in any specimen in the control group, TAP-CSI, and CH-PUI.

## 4. Discussion

This study assessed the effects of CSI, PUI, and Er:YAG laser in the SWEEPS mode for the elimination of TAP, DAP, and CH on PBS of NeoMTA2 CS-based cement to root dentin. The null hypothesis of the study was that no significant difference would be found in the effects of different irrigation methods on PBS of NeoMTA2 to root dentin. In the present study, TAP and DAP were used at a low concentration recommended by the American Association of Endodontists for RETs. CH was also used with a low concentration according to Khoshkhounejad et al. [9]. In the current study, NeoMTA2 was chosen as a CS-based cement devoid of bismuth oxide (which is used as a radio-opacifier in the composition of some CS-based cements) since bismuth oxide can cause severe tooth discoloration [30] and also has an adverse effect on the function and viability of dental pulp stem cells [31–33]. In addition, the presence of tantalum oxide in the composition of NeoMTA2 enhances the formation of mineralized nodules required for pulp regeneration [34]. NeoMTA2 can also lead to the production of CH and release of calcium ions, which are imperative for cell attachment, migration, differentiation, and proliferation and induction of mineralization [35]. It has been found that NeoMTA2 has higher bond strength than Biodentine and ProRoot MTA [36]. The present results showed the highest PBS in the no-medicament control group. Except for the CH-SWEEPS, which had no significant difference with the control group, all other experimental groups had a significantly lower PBS than the control group. This finding may be attributed to incomplete elimination of medicaments from the root canal system by the tested irrigation protocols. According to Alsubait et al. [13], residual medicaments can serve as a barrier and impair complete adaptation of cement to dentinal walls. Besides, low PBS of the medicament groups may be attributed to the chemical effect of the medicaments. For example, CH has a degrading effect on the dentin collagen matrix due to its low molecular weight and alkaline pH [37]. Antibiotic pastes also have a demineralizing effect on dentin due to their acidic nature [38]. Furthermore, shorter contact time of the control specimens with EDTA may be another reason for their higher PBS since long-term exposure to EDTA which is a demineralizing agent can affect dentin microhardness and bond strength of some materials to it by altering the inorganic and organic phases of radicular dentin [39–41].

In CH subgroups of the present study, the elimination of medicament with SWEEPS yielded a higher PBS than CSI and PUI, which may be due to more efficient removal of CH by the SWEEPS technique. Thus, the null hypothesis of the study was rejected. This result was also reported by Kirmizi et al. [18] and Erkan et al. [42]. Also, Usta et al. [43] showed that although complete elimination of CH was not possible with any irrigation method, SWEEPS was significantly more effective than other methods (CSI, PUI, and EDDY) for this purpose. Furthermore, Yang et al. [44] showed lower amounts of CH residues in the SWEEPS and PIPS groups, compared with PUI, especially in the coronal third of the root. These results may be explained by the unique activation mechanism of SWEEPS. In this method, a sudden expansion results in the formation of a second bubble by the second laser pulse, resulting in bursting of the first bubble and formation of a strong wave due to secondary cavitation. The resultant shear stresses and vertical flows cause effective elimination of medicaments, debris, smear layer, and biofilm from the root dentin surface [43, 45]. Lack of a significant difference in PBS of CH-SWEEPS and the control group may indicate more efficient removal of CH by the SWEEPS method.

In the present study, PUI resulted in a higher PBS than CSI in the CH group, which may be due to superior performance of PUI for elimination of CH compared with CSI. Several previous studies also showed the superiority of PUI to CSI for elimination of CH and debris [22, 46, 47].

In TAP and DAP groups, SWEEPS resulted in a higher PBS than CSI and PUI, which may indicate more efficient removal of TAP and DAP by the SWEEPS technique. Usta et al. [43] showed superior cleaning efficiency of SWEEPS for the elimination of DAP compared with other methods (PUI, CSI, and EDDY). Arslan et al. [21] also showed that agitation with PIPS resulted in greater removal of DAP than the CSI. To the best of the authors' knowledge, no previous study is available on the efficacy of SWEEPS for the elimination of TAP from the root canal system; however, Eymirli et al. [19] reported that Er, Cr:YSGG laser-activated irrigation was significantly more effective than the CSI for the elimination of TAP, and Arslan et al. [21] demonstrated that irrigation with PIPS was more effective than EndoActivator and CSI for the elimination of TAP.

In the TAP group of the present study, PUI resulted in a higher PBS than the CSI. Kumar et al. [48] demonstrated that PUI resulted in significantly greater elimination of TAP in comparison with the Canal Brush method. This finding could be attributed to the high flow of the irrigants induced by ultrasonic energy and creation of an acoustic current and cavitation, resulting in superior debris removal from the root dentinal walls [49, 50].

In the DAP group of the present study, the irrigation method (CSI or PUI) did not affect the PBS of NeoMTA2; although Arslan et al. [21] showed that agitation with EndoActivator resulted in greater removal of DAP than the CSI. Pairwise comparisons of the TAP and DAP subgroups in the present study revealed that the irrigation protocol had no significant effect on PBS of NeoMTA2. It may be concluded that the efficiency of all the tested methods for removal of TAP and DAP was such that they produced similar results in terms of PBS of NeoMTA2 to root dentin. Arslan et al. [21] concluded that irrespective of the irrigation method, no significant difference existed between TAP and DAP regarding the amount of residual medicament. Thus, it may be concluded that in the selection of an irrigation method for the elimination of antibiotic pastes, the type of medicament would have no significant effect on the PBS in adoption of the same irrigation protocol. Ghasemi et al. [2] reported similar results, showing that the type of medicament (TAP/DAP) had no significant effect on PBS of MTA and CEM cement while using CSI with 2.5% NaOCl.

Pairwise comparisons of CH with TAP and DAP showed that in both PUI and SWEEPS irrigation techniques, the PBS of CH was higher than that of TAP and DAP, which was in conformity with the previous findings in each subgroup [15, 51, 52]. Kirmizi et al. [18] showed more efficient elimination of CH by the SWEEPS technique; thus, higher PBS in the CH-SWEEPS subgroup of the present study may be attributed to better elimination of CH by the SWEEPS technique. In line with this finding, Arslan et al. [16] reported that the prolonged application of TAP as an intracanal medicament increased the likelihood of bonding and chelation of minocycline with calcium ions, and further complicated its removal. Also, it appears that TAP has a deeper penetration depth and provides greater retention than CH, which is spread on the surface almost superficially; thus, residual TAP can cause dentin demineralization and decrease the pH [37]. Demineralized dentin adversely affects the adhesion of MTA [41]. Thus, more efficient elimination of CH than TAP may be responsible for higher PBS in CH than TAP subgroups. However, Kowsik et al. [29] reported higher PBS of Biodentine in the TAP group compared with CH and attributed it to overdemineralization of the dentin surface by TAP and subsequently greater penetration depth of Biodentine.

On the other hand, Shokouhinejad et al. [41] demonstrated that in low pH environments, the application of unset CH prior to the application of MTA neutralized the acidic pH and increased the PBS of MTA; thus, higher PBS in CSI, PUI, and SWEEPS subgroups of CH in comparison to TAP subgroups may also be attributed to the positive chemical effect of calcium ions on root dentin [41].

The present results showed the adverse effects of medicaments, irrespective of irrigation protocol, on PBS of a CS-based cement to root dentin (except in CH-SWEEPS subgroup); unlike the present study, Topçuoğlu et al. [8] showed that the application of CH and antibiotic pastes had no adverse effects on PBS of MTA, which may be attributed to a difference in methodologies since they used NaOCl and distilled water, while in the present study, EDTA was used for the elimination of medicaments. As mentioned earlier, EDTA may change the chemical structure of dentin and affect dentin adhesion [53].

Assessment of the mode of failure in the present study revealed the dominance of mixed failure in the control, CSI, and PUI groups while cohesive failure had the highest frequency in SWEEPS subgroups.

Aydin studied the bond strength of ProRoot MTA and ERRM putty after the treatment of root dentin with TAP, DAP, and CH. In their study, the most frequently seen failure mode was mixed when no intracanal medicament was applied. Their findings in this regard were in accordance with the results of this study, while their reports about prominence of failure modes in DAP and CH groups were not the same. The difference may be due to different methodologies; since in their study, they used a single protocol for removing all the tested medicaments; while in the present study, different methods were utilized to remove each medicament. Alsubait et al. [13] assessed MTA and BC fast-set putty and reported that the dominance of cohesive failure in the BC group was due to its particle size and uniform particle distribution. Since only one cement type was used in the present study, no comparison could be made. In the present study, the dominance of the cohesive failure mode in the SWEEPS groups may be attributed to more efficient elimination of medicaments by this modality and subsequently better penetration of cement into dentinal tubules. However, the results in the control group were different which calls for further investigations with increased sample sizes.

## 5. Conclusion

In this in vitro study, the bond strength of NeoMTA2 to root dentin could be impacted by many variables including the type of intracanal medicament, the irrigation solutions and protocol, and the opted irrigation technique. Within the study limitations, it appears that CH had a less negative effect on PBS of NeoMTA2 to root dentin in comparison with TAP, irrespective of the irrigation protocol. A higher PBS was also achieved in the CH groups than both TAP and DAP groups in using SWEEPS and PUI. Our findings revealed that when NeoMTA2 was used as the coronal barrier, selecting CH as the intracanal medicament and laser SWEEPS-assisted irrigation protocol would be the preferred method for achieving higher bond strength. Although it must be emphasized that it is not always possible to generalize the results of in vitro studies in the clinical situations considering many limitations such as short incubation period in the study design without considering how results might be affected over time particularly in regenerative treatments that require the coronal seal to be maintained for longer periods. On the other hand, in the clinical scenario, the effect of different removal methods on bond strength of materials to radicular dentin may be affected by other confounding variables such as complexity of root canal anatomy. Further well-designed clinical trials are necessary to be able to recommend a certain protocol for better removing medicaments from root canals in order to achieve a better bond between materials to dentin. We also recommend additional research to investigate the biological and physical mechanisms by which different irrigation techniques may impact bond strength of materials to dentin.

## Figures and Tables

**Table 1:** The means and standard deviations of PBS (MPa) of NeoMTA2 to root dentin in the control and experimental groups.

Medicaments	Irrigation methods		
	CSI	PUI	SWEEPS
CH	6.14 ± 1.49	9.38 ± 2.17	14.21 ± 2.23

DAP	5.95 ± 1.38	7.15 ± 1.67	9.61 ± 1.38

TAP	4.66 ± 1.55	7.34 ± 1.31	10.62 ± 2.23

Control group	15.07 ± 2.51		

**Table 2:** Frequency of different failure modes in the control and experimental groups.

Groups	Failure mode		
	Adhesive	Cohesive	Mixed
Control	0	3 (20%)	12 (80%)
TAP—CSI	0	6 (40%)	9 (60%)
DAP—CSI	1 (0.06%)	5 (33%)	9 (60%)
CH—CSI	3 (20%)	1 (0.06%)	11 (73%)
TAP—PUI	2 (13%)	4 (26%)	9 (60%)
DAP—PUI	2 (13%)	3 (20%)	10 (66%)
CH—PUI	0	7 (46%)	8 (53%)
TAP—SWEEPS	1 (0.06%)	10 (66%)	4 (26%)
DAP—SWEEPS	3 (20%)	7 (46%)	5 (33%)
CH—SWEEPS	2 (13%)	8 (53%)	5 (33%)

## Data Availability

The data that support the findings of this study are available on request from the corresponding author. The data are not publicly available due to privacy or ethical restrictions.
